# Supplementary material for: Predicting visual function by interpreting a neuronal wiring diagram
Source: Nature. 2024 Oct 2;634(8032):113–23. doi: 10.1038/s41586-024-07953-5 (PMC11446822; doi:10.1038/s41586-024-07953-5)
Supplement: Supplementary file 6 — CRF and ERF predictions for individual TmY4 and TmY9 cells. Analogous to Supplementary Data 3, but for TmY target types. Shown are the top four monosynaptic pathways, the strongest pathway passing through each of the top ten intermediary types (ranking from Extended Data Fig. 7), and the trisynaptic pathway Tm1–TmY–Dm3–TmY (see the section entitled Prediction of spatial normalization). [file 41586_2024_7953_MOESM6_ESM.zip › DataS4/TmY9qΓèÑ/720575940628890447.pdf]

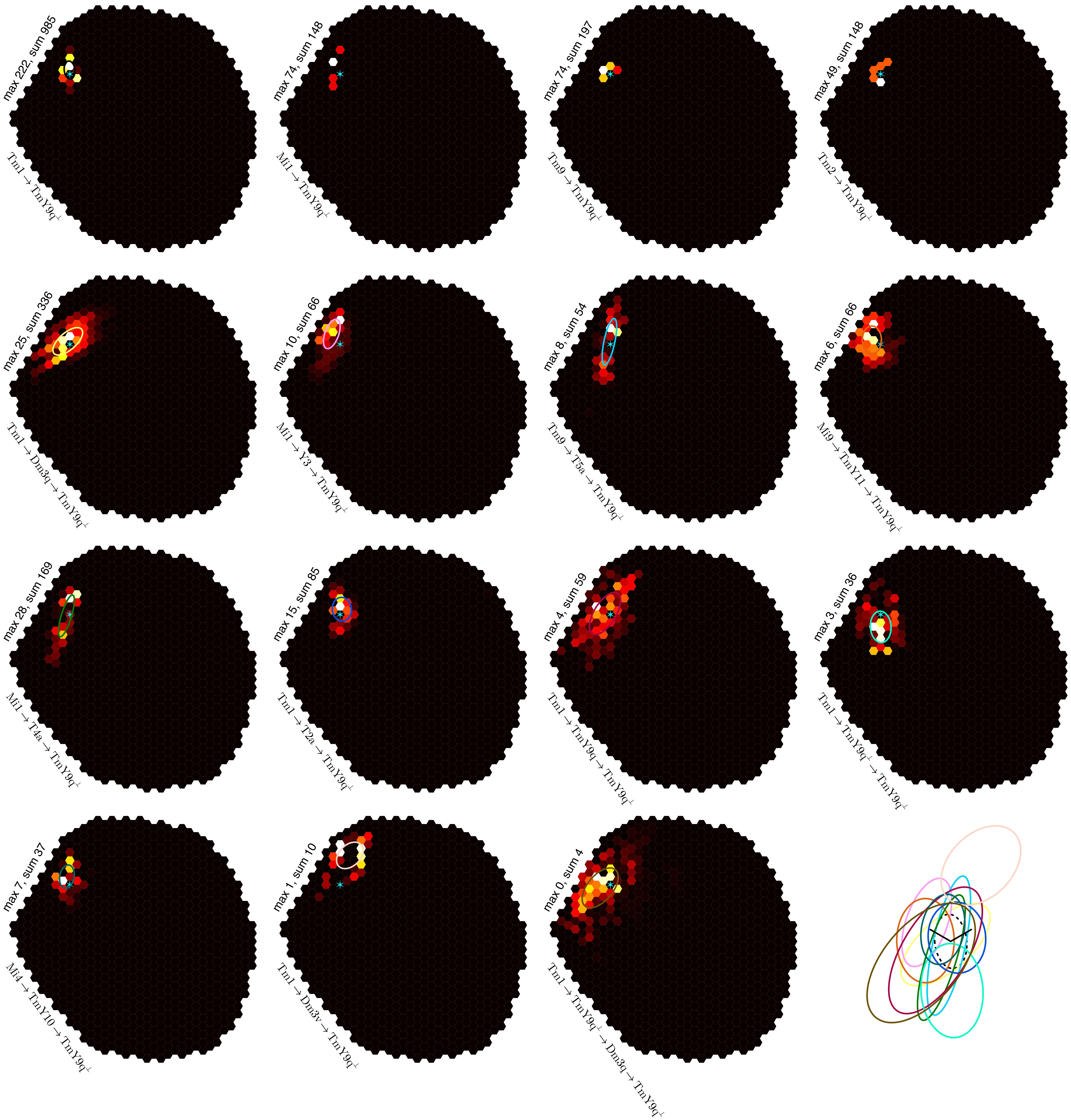
max 222, sum 985
Tm1 → TmY9q⊥
max 74, sum 148
Mi1 → TmY9q⊥
max 74, sum 197
Tm9 → TmY9q⊥
max 49, sum 148
Tm2 → TmY9q⊥
max 25, sum 336
Tm1 → Dm3q → TmY9q⊥
max 10, sum 66
Mi1 → Y3 → TmY9q⊥
max 8, sum 54
Tm9 → T5a → TmY9q⊥
max 6, sum 66
Mi9 → TmY11 → TmY9q⊥
max 28, sum 169
Mi1 → T4a → TmY9q⊥
max 15, sum 85
Tm1 → T2a → TmY9q⊥
max 4, sum 59
Tm1 → TmY9q → TmY9q⊥
max 3, sum 36
Tm1 → TmY9q⊥ → TmY9q⊥
max 7, sum 37
Mi4 → TmY10 → TmY9q⊥
max 1, sum 10
Tm1 → Dm3v → TmY9q⊥
max 0, sum 4
Tm1 → TmY9q⊥ → Dm3q → TmY9q⊥
